# Molecular Evolutionary Analysis of Potato Virus Y Infecting Potato Based on the VPg Gene

**DOI:** 10.3389/fmicb.2019.01708

**Published:** 2019-07-26

**Authors:** Yanzhi Mao, Xuhong Sun, Jianguo Shen, Fangluan Gao, Guangwei Qiu, Teng Wang, Xianzhou Nie, Wei Zhang, Yanling Gao, Yanju Bai

**Affiliations:** ^1^Heilongjiang Academy of Agricultural Sciences, Harbin, China; ^2^Inspection and Quarantine Technology Center, Fujian Exit-Entry, Inspection and Quarantine Bureau, Fuzhou, China; ^3^Institute of Plant Virology, Fujian Agriculture and Forestry University, Fuzhou, China; ^4^Fredericton Research and Development Centre, Agriculture and Agri-Food Canada, Fredericton, NB, Canada

**Keywords:** potato virus Y, viral genome linked protein, Bayesian phylogenetic analysis, selective constraint, Bayesian tip-association significance

## Abstract

Potato virus Y (PVY) is an important plant pathogen infecting solanaceous crops, causing significant losses to global potato and tobacco production. Some aspects of the plant pathology and molecular biology of PVY have been studied intensively, but the evolutionary dynamics of this virus are poorly understood. Here, we performed a comprehensive set of rigorous evolutionary analyses using 177 nucleotide sequences of the viral genome linked protein (VPg) gene, which interacts with the plant eukaryotic translation initiation factor 4E (eIF4E). Our Bayesian analysis reveals that the VPg gene of PVY has been evolving at a rate of 5.60 × 10^–4^ subs/site/year (95% credibility interval 3.35 × 10^–4^–8.17 × 10^–4^), which is equivalent to those of other plant-infecting RNA viruses. We identified different evolutionary constraints on the two clades of PVY, clade N and clade O, whose diverge time were estimated at the year 1861 CE (95% credibility interval 1750–1948 CE). We also found that genetic variations were correlated with geographic regions, suggesting that the evolution of this pathogen is strongly affected by geographical associated factors. Taken together, the results of our study have potential implications for the control strategies of PVY.

## Introduction

Potato virus Y (PVY), the type member of the genus *Potyvirus* (Lefkowitz et al., 2018), is one of the most destructive plant pathogens causing considerable economic losses in *Solanaceae* crops. It has become one of the most prevalent viruses of potato and tobacco productions worldwide (Karasev and Gray, 2013; Quenouille et al., 2013). PVY exists as different strain groups, including the conventional PVY^O^ and PVY^N^ strains and the newly emerged recombinant strains such as European (Eu)-PVY^NTN^, PVY^N:O^, PVY^N–Wi^, and PVY^NTN–NW^ (Karasev and Gray, 2013; Bai et al., 2019). The classic strains (i.e., PVY^O^ and PVY^N^) are defined according to their reactions in potato cultivars bearing different *N* genes and in common tobacco (Singh et al., 2008). PVY^O^ and PVY^C^ induce hypersensitive responses (HR) in cultivars bearing the *Ny* and *Nc* genes, respectively, whereas PVY^N^ does not induce HR in cultivars harboring these genes. In contrast, PVY^N^ elicits the classic veinal necrosis on common tobacco plants whereas PVY^O^ and PVY^C^ only cause mosaic symptoms on the plants (Singh et al., 2008; Karasev and Gray, 2013). Most of the newly emerged strains were derivatives of PVY^O^ and PVY^N^ via genome recombination (Singh et al., 2008; Karasev and Gray, 2013; Nie et al., 2013). These strains possess one to four recombinant events (RE) (Green et al., 2017). For instance, in PVY^N:O^, one RE located at ca. ∼nt 2,400 nt (N to O) can be found; whereas in Eu-PVY^NTN^, three REs at ∼nt 2,400 nt (N to O), ∼nt 5,820 (O to N) and ∼nt 9,180 (N to O) can be found (Nie et al., 2004). PVY induces various foliar symptoms, ranging from mosaic, mottling, lesion to stunting and necrosis on potato. The severity of symptoms depends on the potato varieties, the type of virus strain and environment conditions. It is particularly noteworthy that PVY^NTN^ not only induces symptoms on foliage, but also causes a severe tuber condition termed potato tuber necrotic ringspot disease in sensitive cultivars (Nie et al., 2012, 2013). In nature, PVY is mainly transmitted horizontally from plants to plants by a number of aphid species in a non-persistent manner, and vertically through infected potato seed tubers (Lacomme et al., 2017). Mechanical transmission caused by management practices (e.g., tractor movement during pesticide applications) has also been reported (MacKenzie et al., 2018).

PVY consists of a positive-strand RNA genome ∼9.7 kb in size and encodes a polyprotein of 3061 amino acids (Lefkowitz et al., 2018). The polyprotein is cleaved by three virus-encoded proteinases to yield up to 10 mature proteins (Adams et al., 2005), in which the viral protein genome-linked (VPg) is an intrinsically disorder protein (Grzela et al., 2008) and has been shown to interact with a variety of partners, including itself, allowing it to participate in RNA replication, virus movement, translation, RNA-silencing suppression, and phloem loading of the virus (Quenouille et al., 2013). The free VPg is linked covalently to the 5′-terminus of the genomic RNA and is required for potyvirus infectivity during the virus life cycle (Eskelin et al., 2011; Ivanov et al., 2014). Interestingly, a central intrinsically disordered region (IDR) localized between the amino acid positions 95 and 120 in VPg is a key determinant of PVY adaptation to the eukaryotic translation initiation factor 4E (eIF4E)-based recessive resistance against the virus (Moury et al., 2004, 2014). The contribution of intrinsic disorder to VPg-mediated PVY adaptation has recently been evaluated (Charon et al., 2018).

As one of the top 10 most famous plant viruses (Scholthof et al., 2011), some aspects of the plant pathology, molecular biology and population genetics of PVY have been studied intensively (Lorenzen et al., 2006; Chikh-Ali et al., 2013; Kim et al., 2016; Gao et al., 2017; Bai et al., 2019). However, few studies have focused on the evolutionary dynamics and timescale of PVY. A previous review suggested that PVY group of viruses primarily diverged in the Americas approximately 2400 years ago (Gibbs and Ohshima, 2010). Using Bayesian molecular dating the parental strains of PVY were dated to the period of the first introduction of potatoes to Europe (Visser et al., 2012). This is consistent with the recent origins inferred for various plant viruses (Gibbs et al., 2010). However, studies of the more recent evolutionary dynamics of PVY remain limited, especially at the global level, although such information is vital for designing strategies and management schemes. In this study, we use a Bayesian phylodynamics framework to infer the evolutionary rate and the time to recent common ancestor (tMRCA) based on the VPg gene sequences of PVY isolates collected from the main producing regions in China, combined with all published sequences in GenBank. We also investigated the correlation between the genetic variation and geography during PVY evolution.

## Materials and Methods

### Sampling and VPg Gene Sequencing

A total of 44 PVY isolates from potato (*Solanum tuberosum*) across the main potato producing regions in China including Heilongjian, Hunan, and Hebei provinces between 2011 and 2017 were randomly collected (Supplementary Table S1). Leaf samples infected with PVY were stored at −80°C for later use. The PVY-infected tuber samples were kept in a greenhouse as described in Han et al. (2017), and the infected *in vitro* plantlets were maintained in a tissue culture room.

Total RNA was extracted from leaf tissue using Trizol reagent and was reverse transcribed according to the manufacturer’s instructions (Invitrogen, Carlsbad, CA, United States). The VPg gene was amplified using two primers designed from highly conserved regions of PVY genomes, as descripted by Gao et al. (2014). PCR amplifications were conducted in a total volume of 50 μL containing 2 μL of template cDNA, 10 μL 5 × PrimeSTAR Buffer (Mg^2+^ Plus), 4 μL of dNTP Mixture (2.5 mM each), 1.5 μL of forward primer (5′-GGYTCTGCATGYGAGGARAAT-3′) and 1.5 μL of reverse primer (5′-CAAACTGTTTGRGCAATTGGRTT-3′), 0.5 μL of PrimeSTAR HS DNA Polymerase (2.5 U/μl), and 30.5 μL of ddH2O. The PCR program conditions were as follows: after initial denaturation step at 94°C for 3 min; 35 cycles were performed consisting of three steps: denaturation at 94°C for 30 s, annealing at 50°C for 30 s, and extension at 72°C for 50 s. The final elongation step was performed at 72°C for 5 min. PCR products were electrophoresed on 1.0% agarose gels in Tris–acetate-EDTA (TAE) buffer and visualized under UV illumination after staining with ethidium bromide (0.5 μg/mL). PCR products were purified using an QiAquick Gel Extraction kit (TransGen, Beijing), ligated into the pMD18-T vector (TaKaRa, Dalian), and transformed into *Escherichia coli* strain DH5α cells. The recombinant plasmids were purified and at least three cDNA clones were sequenced to ensure consensus in both directions by Sangon Biotech (Shanghai) Co., Ltd.

### Data Set

Host-driven adaptation could affect the diversification of viral isolates (Cuevas et al., 2012b). Only PVY sequences derived from potato were included in this study. In addition to 44 novel sequences, 133 published VPg sequences derived from potato were retrieved from GenBank database to give a total data set consisting of 177 VPg sequences (Supplementary Table S1). The isolates were collected from 15 countries between 1963 and 2017 with known sampling dates and geographic regions (Supplementary Figure S1). The sampling regions were Africa (*n* = 11), Asia (*n* = 74), Europe (*n* = 17), North America (*n* = 73), and South America (*n* = 2). We constructed a codon-based alignment for the sequences using the MAFFT algorithm (Katoh and Standley, 2013) within TranslatorX online server with the default settings (Abascal et al., 2010).

### Recombination Analysis

To investigate the role of recombination in PVY evolution, we constructed recombination analyses using two different approaches to identify potential recombination events in VPg sequences. Firstly, we performed a split-decomposition network analysis and calculated the pairwise homoplasy index using the neighbor-net algorithm in SplitsTree 4.13.1 (Huson, 1998). We subsequently identified potential recombinant and parental sequences using seven different algorithms, including RDP, GENECONV, BOOTSCAN, MAXCHI, CHIMAERA, SISCAN, and 3SEQ, implemented in the RDP 4.95 packages (Martin et al., 2015). Default settings were used throughout, except that the highest acceptable *p*-value cut-off was set to 0.01 with a standard Bonferroni correction. To avoid misidentification of recombination, recombination events were only considered to be significant when they were simultaneously supported by at least four of the seven algorithms with *p*-value < 10^–6^.

### Tests for Temporal Signal

The standard Bayesian dating permutation test for temporal signal can be seriously misled for data sets in which temporal and genetic structures are confounded (Murray et al., 2016). To exclude this possible effect on molecular dating, we first performed a Mantel test of the correlation between pairwise genetic distances and differences in sampling dates and calculated the *p*-value of this test with 1000 permutations. We found evidence of confounding of temporal and genetic structure in the data (*P* = 0.001, Supplementary Figure S2A), so we applied the clustered permutation test introduced by Duchêne et al. (2015), which was recommended by Murray et al. (2016), to confirm the presence of temporal signal. In this test, a data set has sufficient temporal signal when the mean rate estimate from the original data set falls outside the 95% credibility intervals of the rate estimates from 10 clustered date-randomized replicates.

### Bayesian Phylogenetic Analysis

To analyze the evolutionary rate and time scale of PVY, we applied a Bayesian framework for inference as in BEAST 1.8.4 (Drummond et al., 2012). The sequences were analyzed using the HKY + *G*_4_ substitution model, which was selected using ModelFinder implemented in IQ-Tree 1.5.5 (Nguyen et al., 2014), based on Bayesian Information Criterion (BIC). We further confirmed the adequacy of the selected substitution model for our data set using PhyloMAd (Duchêne et al., 2018; Supplementary Figure S3). The best-fit clock model (the strict and relaxed clocks) and the best-fit tree prior (among the constant size, exponential-growth, and Bayesian skyline coalescent), were selected using marginal likelihoods estimated by path sampling (Baele et al., 2012). An uncorrelated lognormal relaxed clock and Bayesian skyline coalescent tree prior provided the best fit for our data set (Supplementary Table S2). Isolation time of viral sequences were used to calibrate the molecular clock. Four independent Markov Chain Monte Carlo (MCMC) analyses were run simultaneously for 100 million generations, with samples drawn every 10,000 generations. Convergence of all parameters was verified visually using Tracer 1.7 (Rambaut et al., 2018).

In addition to Bayesian rate estimate, we employed an approximate maximum likelihood approach implemented in TreeTime (Sagulenko et al., 2018) to infer the evolutionary rate of the VPg gene using a regression of phylogenetic root-to-tip distances against sampling date.

### Phylogeny-Geography Association and Population Structure Analyses

To assess the association between phylogeny and the pattern of the geographical structure of PVY, we used the software BaTS 2.0 (Parker et al., 2008) to calculate values of association index (*AI*), parsimony score (*PS*), and monophyletic clade (*MC*) size statistics from the posterior sample of trees produced by BEAST, as described above. This method accounts for phylogenetic uncertainty in investigating phylogeny-trait correlations, with 1000 random permutations of tip locations to estimate a null distribution for each statistic. Low *AI* index and *PS* and high *MC* scores suggest a strong phylogeny–geography association and low spatial admixture. The ratio of the observed to expected mean association index was also computed, as a measure of the strength of the association between the geography and the phylogeny. This ratio ranges from 0, indicating complete population subdivision, to 1, suggesting an unstructured population. Since the newly collected 44 PVY isolates were sampled within a relatively narrow time window (2011–2017), we used a bootstrapping approach to standardize sample sizes and performed analyses of 10 replicate subsamples to minimize the influence of sampling biases in our data set. For each bootstrap replicate, we randomly sampled 11 sequences without replacement from each geographic region.

We also used the package *adegenet* implemented in R 3.5.1 to investigate the genetic population structure present among geographic regions using a discriminant analysis of principal components (DAPC), which does not rely on assumptions of Hardy Weinberg Equilibrium and panmixia (Jombart et al., 2010). The PVY population of South America was excluded from analyses due to an inadequate sample size.

### Analysis of Selection Pressures

The inference of selection was performed using the branch-specific models (Yang and Nielsen, 1998, 2002) of the CODEML algorithm (Yang, 2007) implemented in EasyCodeML (Gao et al., 2019). For the branch model, the ratio of non-synonymous vs. synonymous substitutions (ω = *d*N/*d*S) under two *a priori* assumptions: one-ratio model assuming that the entire tree has been evolving at the same rate and two-ratio model allowing foreground branch to evolve under a different rate. We used likelihood ratio test to verify which of the models best fit the data by comparing twice the difference in log-likelihood values between pairs of the models using a *x*^2^-distribution, with the degrees of freedom equal to the differences in the number of parameters between the models (Yang, 1998). We also applied the false discovery rate (FDR) method to performed multiple testing correction (Storey and Tibshirani, 2003) implemented in R. Only a *p*-value of less than 0.05 in the likelihood ratio test was considered to be significant. As an additional check on the sensitives test for the branch-specific model, we estimated pairwise *d*N/*d*S ratios using the yn00 program in PAML (Yang and Nielsen, 2000). For more-inclusive analysis, the site models were used to detect signatures of positive selection in VPg using EasyCodeML (Gao et al., 2019). We performed likelihood-ratio test to compare the fit of three nested models, including M3 vs. M0, M2a vs. M1a, and M8 vs. M7. When these tests yielded a significant result (*p* < 0.01), we then used the Bayes empirical Bayes method to identify individual positively selected codon sites (posterior probability > 0.95).

## Results

### Sequencing of PVY in China

**FIGURE 1:**
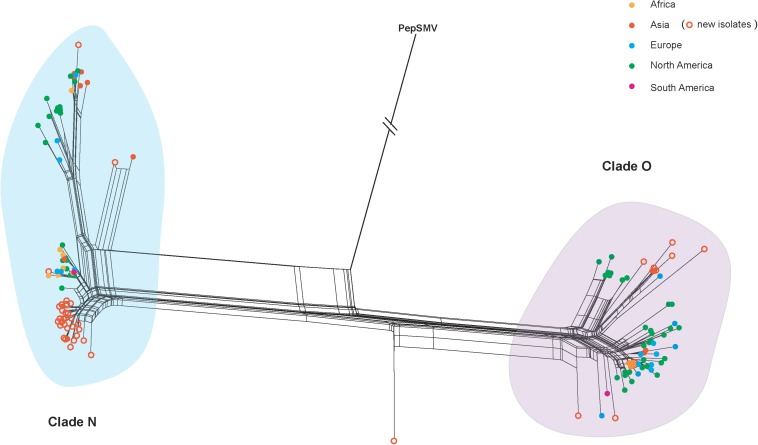
Splits network analysis of the VPg gene from 177 potato virus Y isolates from different geographic regions. An isolate of pepper severe mosaic virus (accession number: NC_008393) was used as an outgroup. Colors indicate isolates from different geographic regions. The scale bar is the genetic distances. PVY isolates newly sequenced in this study are marked with empty circle in orange.

The VPg gene sequences obtained in this study have been deposited in GenBank with accession numbers MK144421-MK144464. Using the SplitsTree program, we obtained a split network that showed reticulate topologies, indicating several potential recombination events among the PVY isolates (Figure 1). The pairwise homoplasy index also consistently identified a significant signal of recombination in our data set (*p* = 7.82 × 10^–4^). Using the RDP4 suites, one likely recombination event in the PVY-(accession number: MK144459) was identified as recombinant at high level significance by four algorithms (GENECONV, *p* ≤ 4.26 × 10^–9^; MAXCHI, *p* ≤ 2.31 × 10^–8^; CHIMAERA, *p* ≤ 7.68 × 10^–8^; and 3SEQ, *p* ≤ 2.9 × 10^–12^). Since including recombinants in evolutionary analysis may result in incorrect estimates of the rate of evolution (Sironi et al., 2015), this recombinant was removed. All recombination-free VPg sequence data from PVY isolates, even those that have combined recombination structure based on whole genome, were used for the subsequent phylogenetic dating and phylodynamic analyses.

### Identification of Evolutionary Constraints

**FIGURE 2:**
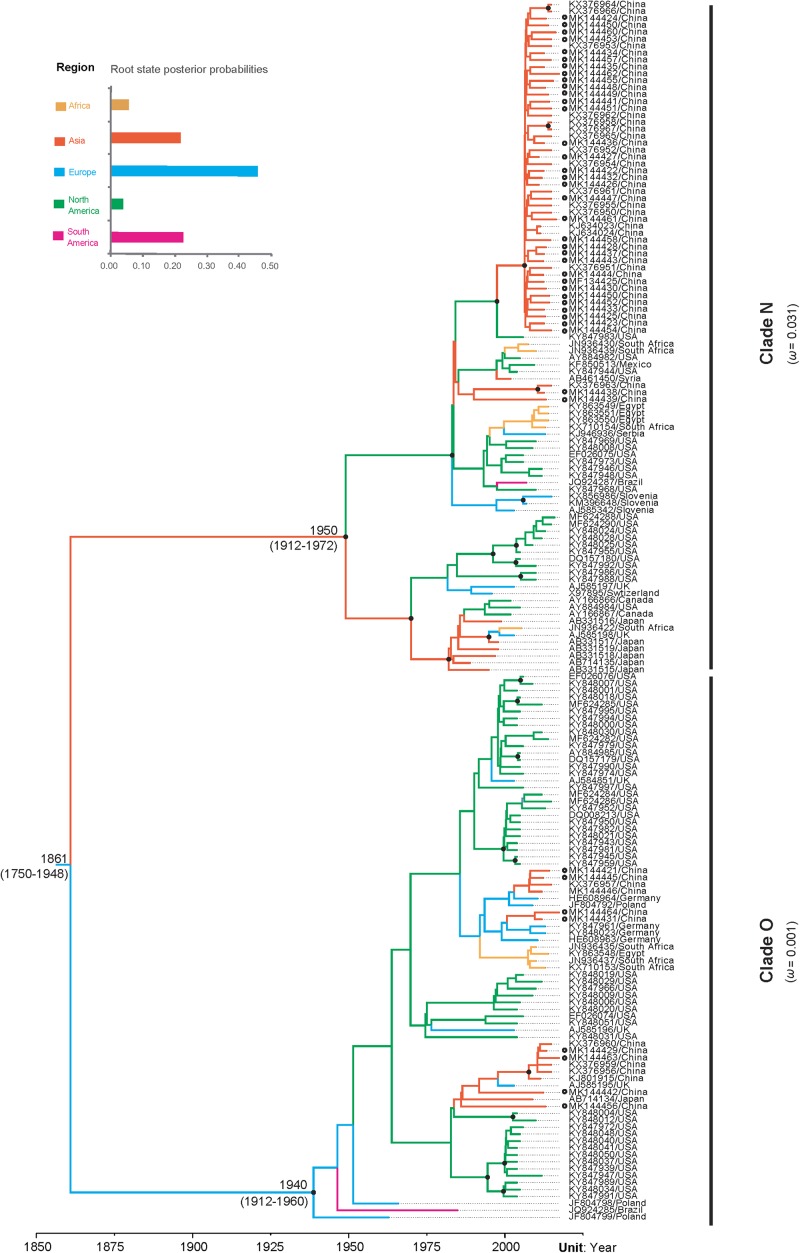
The maximum clade credibility tree inferred by Bayesian analysis of sequences of the VPg gene of potato virus Y and comparison of *d*N/*d*S values between the two clades. Bayesian estimates of divergence time, upper and lower limits of the 95% highest posterior density (HPD) estimates are shown for major nodes. Black dots indicate strong node support (posterior probability ≥ 95%). Branch lengths are scaled in units of time, as indicated by the time axis. Branch colors denote inferred location states, as shown in the color key. The root state posterior probabilities estimated for each geographic region are shown in the inset panel. PVY isolates newly sequenced in this study are marked with empty circle.

Our time-scaled maximum clade credibility (MCC) tree indicated that PVY isolates could be clustered into two distinct clades, called clade N and clade O (Figure 2). In despite of a certain degree diversity in sampling regions, most PVY isolates tended to cluster according to their geographic origin, either within clade N or clade O. The overall ω estimates were 0.031 for the clade N and 0.001 for the clade O, respectively (Supplementary Table S3 and Figure 2). This result indicates that clade N evolved under reduced purifying selection compared with clade O. Furthermore, similar results obtained from pair-wise analyses showed that there are differences between the distribution of *d*N/*d*S values between clade N and clade O (Supplementary Figure S4). Using the site model, purifying selection was detected at the majority of polymorphic sites (Supplementary Figure S5). Positive selection was also detected at amino acid positions 96 and 164, but without significant (>0.95) posterior probability (Supplementary Figure S5).

### The Phylodynamic Patterns and Demographic History of PVY

Our data set passed the date-randomization test (DRT) that showed no overlaps between the true estimate of evolutionary rate and 95% credibility interval generated from 10 replicates of randomized data sets (Supplementary Figure S2B). This suggests that the data set has adequate temporal signal for reliable Bayesian tip-dated analyses.

**FIGURE 3:**
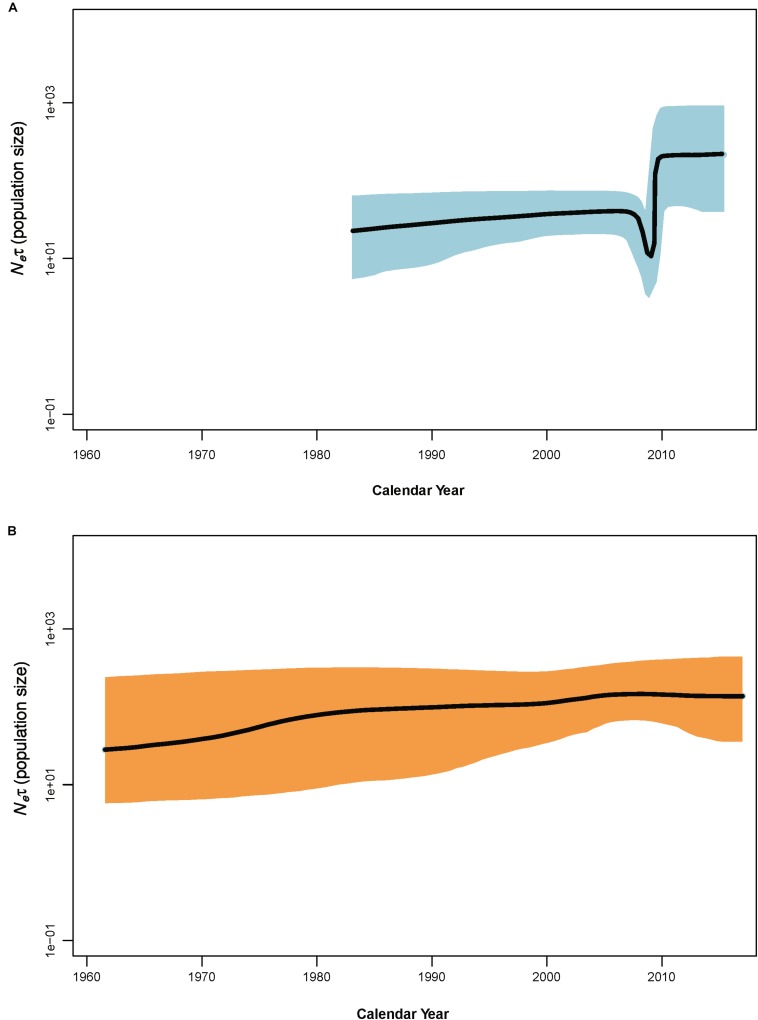
Bayesian skyline plots of population dynamics for clade N **(A)** and clade O **(B)** of potato virus Y. The *x*–axis gives units of years, and *y*–axis is a measure of relative genetic diversity. The shade areas in light blue are within the 95% highest posterior density interval. The dotted vertical lines indicate important population-size changes for potato virus Y. The shorter timescales of the clade N skyline plots are due to the shorter sampling period.

The results of our Bayesian phylogenetic analysis suggested that PVY probably emerged in Europe (root posterior probability = 0.46), with a recent common ancestor in 1861 CE (95% credibility interval 1750–1948 CE). The most recent common ancestor of the PVY^N^ strain was located at approximately 1940 CE (95% credibility interval: 1912–1972 CE), whereas the most recent common ancestor of PVY^O^ strains was 1950 CE (95% credibility interval: 1912–1960 CE) (Figure 3). Our Bayesian estimated mean substitution rate was 5.60 × 10^–4^ subs/site/year (95% credibility interval 3.35 × 10^–4^–8.17 × 10^–4^), whereas the root-to-tip regression method obtained an estimate of 8.73 × 10^–4^ subs/site/year.

Coalescence-based Bayesian skyline plot (BSP) revealed an explicit demographic history for the PVY populations of clade N and clade O (Figure 3) – showing that the population size of PVY from clade N suffered from a decline in size in 2006–2007 prior to a period of slight increase and then experienced a sudden population expansion approximately in 2009, followed by a period of stability from 2010 to the last sampling year, whereas the population size of PVY from clade O remained relatively constant throughout the past decades.

### The Patterns of Geographic Structure of PVY

Although the MCC tree for the VPg gene seem to not show a clear geographic structure (Figure 2), the global trait association tests of phylogeographic structure rejected the null hypothesis of no association between the geographic and phylogenetic relationship (*AI*, *p* < 0.001 and *PS*, *p* < 0.001, Table 1). Significant population subdivision was observed for four geographic regions either in original data set or among the 10 replicate subsamples when the *MC* statistic was shown (Table 1 and Supplementary Table S4). This indicates strong geographic structure in PVY, suggesting the diversification of the virus is explained by geography-driven adaptation. We also found that the index ratios of the observed values to those expected were between 0 and 1 among geographic regions, with association indices of 0.12 (95% credibility interval: 0.07–0.20) and parsimony scores of 0.33 (95% credibility interval: 0.27–0.39), suggesting the evolution of PVY is accounted for a geographic structure but not homogeneous.

**TABLE 1:** phylogeny–trait association analysis for the phylogeographic structure of potato virus Y using Bayesian tip-association significance testing.

**Statistic**	**# Isolates**	**Index ratio (95% HPD CIs)**	**Observed mean (95% HPD CIs)**	**Null mean (95% HPD CIs)**	***P*−value**
Association index		0.12 (0.07–0.20)	1.53 (0.91–2.24)	12.27 (11.06–13.25)	< 0.001
Parsimony score		0.33 (0.27–0.39)	25.42 (22.00–29.00)	77.92 (74.19–81.36)	< 0.001
**Maximum monophyletic clade**					
Asia	73	n/a	48.21 (48.00–50.00)	3.48 (2.87–4.39)	0.010
Europe	17	n/a	2.83 (2.00–4.00)	1.44 (1.08–2.01)	0.010
North America	73	n/a	13.79 (11.00–17.00)	3.43 (2.89–4.32)	0.010
Africa	11	n/a	3.97 (3.00–4.00)	1.23 (1.01–2.00)	0.010
South America	2	n/a	n/a^*^	n/a^*^	n/a^*^

Index ratio, observed mean/null mean; HPD CIs, highest posterior density confidence intervals; n/a, no data available;*, *insufficient sample size (n < 3).*

**FIGURE 4:**
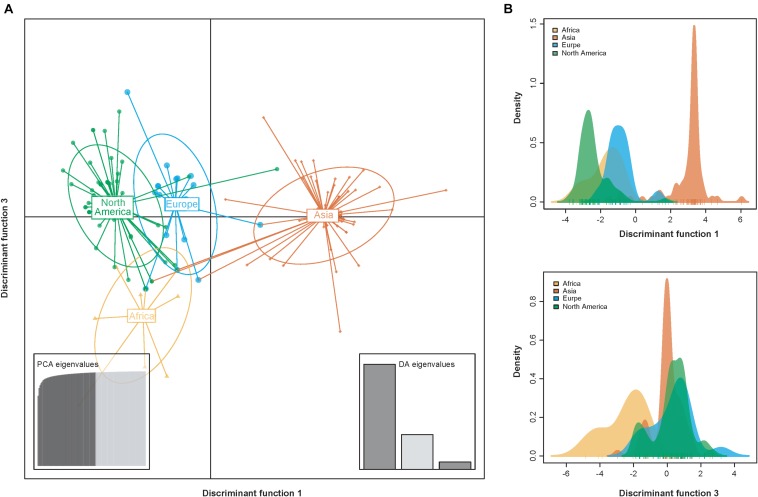
Scatterplots resulting from the discriminant analysis of principal components (DAPC). **(A)** Individual isolates from the same geographic regions are depicted as unique color shapes and surrounded by 95% inertia ellipses. The PCA and DA eigenvalues inset panels show the overall variability among individuals and the relative capture of variance for each discriminant function, respectively. The *y*- and *x*-axes respectively indicate the first and third discriminant principal components, which best summarize the differences between clusters while neglecting within-cluster variation. **(B)** The discriminant functions for separating the clusters.

Consistent with the results from the phylogeny- geography association analysis (Table 1), DAPC scatter plots also indicated that the Asia population were relatively distinct from the other populations along the first discriminant function axis, while the Africa population exhibited more subtle structure along the third discriminant function axis (Figure 4). This suggests that geography contributes to population differentiation among PVY populations.

## Discussion

Overall, we performed a large-scale phylodynamic analysis of the global population of PVY using newly sequenced and curated sequence data retrieved from GenBank. Previous works have found evidence that recombination is an important driving force in the evolution and divergence of many plant viruses, including potyviruses (Nagy, 2008). However, only one recombinant was identified in our analysis. One plausible explanation for this observation is strong selective pressure against survival of new PVY recombinants. Consistent with this possibility, our selective analyses suggested that the VPgs were under strong purifying selection within both clades (0 < ω < 1, Figure 2). Another explanation is that the genomic segment encoding VPg is not a hot spot for recombination. This explanation is consistent the results obtained by Green et al. (2017), which show that recombinant junctions are not frequently seen in the genomic segment corresponding to VPg.

Although PVY exists as complex strain groups, most potato-infecting PVYs are thought to be recombinants of PVY^O^ and PVY^N^. Consistent with this, our Bayesian phylogenetic analysis resolved VPgs into two large clades, with VPgs from well-characterized PVY^O^ (such as the representative isolate of PVY^O^ Oz, accession number EF026074) and PVY^N^ strains (such as the N605 isolate, accession number X97895) in either of the two clades. Like that observed in by Cuevas et al. (2012a), we also found that VPgs in clade O (mostly likely derived from PVY^O^ strain) was subjected to stronger purifying selection than those in clade N (mostly likely derived from PVY^N^ strain). A possible explanation for the difference is that genes evolving under relaxed selective constraints may more readily adopt new forms of biased expression during the evolution of alternate phenotypes (Hunt et al., 2011). As expected, PVY^O^ strains can induce severe leaf mosaic symptoms on potato, whereas PVY^N^ strains are reported to induce mild mosaic or mottle in leaves but not severe symptoms in potato plants (Quenouille et al., 2013). Cuevas et al. (2012b) detected a positive selection site in the central region of VPg. This site was not found in our study. However, we did find two positive selection sites in the vicinity of this site, although both of which were not statistically significant (with posterior probabilities < 80%). The fact that two independent studies, using different datasets and with different methods, detected positive selection in the central region of VPg is intriguing. A hypothesis that deserve further studies is that the central region of VPg may be involved in its physical interactions with eIF4E.

Our Bayesian phylogenetic analysis placed the root of the tree in Europe with strong supports (Figure 2). This coincides with previous studies that PVY was probably introduced to Europe by potato tubers taken from South America that subsequently seeded other regions of the world after the second half of the nineteenth century. However, there is no evidence that it is transmitted by sexually produced seed (Visser et al., 2012; Gibbs et al., 2017). In addition, our analysis dated the N and O clade in the year 1861 CE (95% credibility interval 1750–1948 CE). This timeframe is similar to that estimated by Visser et al. (2012), but older than that reported by Gibbs et al. (2017), which placed the most recent common ancestor around 1000 CE. These discrepancies might be due to the result of sampling differences, in which we excluded the early Chilean isolates that were first to diverge during the PVY evolution (Quenouille et al., 2013).

The estimated substitution rate of the VPg gene from our Bayesian analysis is 5.60 × 10^–4^ subs/site/year (95% credibility interval 3.35 × 10^–4^–8.17 × 10^–4^). This is similar to our estimate of 8.73 × 10^–4^ subs/site/year from a regression of root-to-tip genetic distances against the sampling dates. Several previous studies have shown that the estimates of substitution rates might be unreliable if there is strong population structure (Navascues and Emerson, 2009). In that case, the clustered permutation approach of Duchêne et al. (2015) can still give reliable results, regardless of whether it is applied to linear regression or Bayesian dating (Murray et al., 2016). In combination with the results of the clustered permutation test (Supplementary Figure S2B) in this study, this provides support for the substitution rate estimated from our Bayesian analysis to be reliable, although confounding of temporal and genetic structure is present in our data set (Supplementary Figure S2A).

Our demographic analyses found that PVY experienced obvious population expansions during the past 30–40 years. However, this expansion was interrupted approximately in the year 2006 (Figure 3). Such a demographic history of PVY coincides well with the history of potato production in the word. Based on FAO data^1^, the cultivation area of potato increased from ∼20.91 million ha in 1985 to ∼23.63 million ha in 2005, and from ∼23.89 million ha in 2009 to ∼24.62 in 2016. However, the period from 2006 to 2008 saw a slight decrease in potato cultivation (from ∼23.63 million ha in 2005 to ∼22.01 million ha in 2006). This coincidence suggested a direct impact of potato cultivation on the population size of PVY. Further studies aiming to understand how potato cultivation area influences the population size of PVY will be interesting.

Genetic variability of many potyviruses is related to the geographical origin of the viral isolates, including chili veinal mottle virus (Gao et al., 2016), papaya ringspot virus (Gibbs et al., 2008) and telosma mosaic virus (Xie et al., 2017). Similar observations have been found in PVY, where significant spatial structure in the differentiation of PVY population, on either a global scale or national scale, has been observed when using the CP sequences (Cuevas et al., 2012a; Gao et al., 2017). No surprisingly, the results obtained in this study have also shown a clear correlation between the phylogeny and the geographical origins (Table 1 and Figure 4), providing further evidence that geographically driven adaptation is an important determinant of PVY differentiation.

## Conclusion

In summary, the results obtained in our study shows that phylodynamic analysis of the VPg gene data can shed light on the temporal dynamics of PVY. We found that genetic variations of this pathogen were correlated with geographic regions. It will also be important to study human-mediated dispersal in the global population of PVY, which will need to be further analyzed, which will lead to a more comprehensive picture of its evolution.

## Data Availability

The datasets generated for this study can be found in NCBI, MK144421-MK144464.

## Author Contributions

YB and XN conceived the study. YM, XS, GQ, TW, WZ, and YG performed the experiments. YB, FG, JS, and XN analyzed the data and interpreted the results. FG and YB led the writing of the manuscript. All authors contributed to the manuscript and agreed on the manuscript before review.

## Conflict of Interest Statement

The authors declare that the research was conducted in the absence of any commercial or financial relationships that could be construed as a potential conflict of interest.

## Footnotes

1 http://www.fao.org/faostat

## Supplementary Material

FIGURE S1 Map showing the localities of potato virus Y isolates included in this study. The *ggplot2* and *maps* libraries in *R* 3.5.1were used to create the map. Novel viral isolates obtained in this study are indicated by black dot.

FIGURE S2 Results of tests for the presence of temporal signal in the sequence data. **(A)** Mantel test of confounding of genetic and temporal distances. The *y*-axis shows differences in sampling years, and the *x*-axis indicates genetic distance. **(B)** Date-randomization test using clustered permutations. The *y*-axis indicates the substitution rate on a log_10_ scale, and the *x*-axis shows the actual data set and 10 different clustered permutations of the dates in the data set. The dashed red lines indicate the 95% credibility interval of the rate estimate from the actual data set.

FIGURE S3 Test of substitution model adequacy for the HKY + *G*_4_ substitution model, as assessed using five test statistics available in PhyMad: **(A)** chi-squared statistic, **(B)** multinomial likelihood, **(C)** biochemical diversity, **(D)** consistency index, and **(E)** squared Mahalanobis distance based on the first four statistics. The histograms of the values of the test statistics are calculated from simulation data set, with the red line indicating the value for the empirical data set. The tail-area probability, empirical test statistic, and standard deviations of the predictive distribution (SDPD) are shown in the top-right of each panel.

FIGURE S4 Comparison of *d*N/*d*S values between the two clades. Boxplots showing the dN/dS ratio of clade O and clade N for the VPg gene.

FIGURE S5 Sliding window plot of *d*N*/d*S ratios for the VPg gene. Sites under neutral (*d*N/*d*S = 1) are indicated in red dotted line. The window size is 20 codons, and the offset between windows is one codon.

TABLE S1 Isolates of potato virus Y used in this study.

TABLE S2 Marginal likelihoods of different combinations of clock model and tree prior.

TABLE S3 Selective constraint acting on the VPg gene.

TABLE S4 Results of subsampling-Bayesian tip-association significance testing.
