# Supplementary figures and images for: Molecular Evolutionary Analysis of Potato Virus Y Infecting Potato Based on the VPg Gene
Source: Front Microbiol. 2019 Jul 26;10:1708. doi: 10.3389/fmicb.2019.01708 (PMC6676787; doi:10.3389/fmicb.2019.01708)

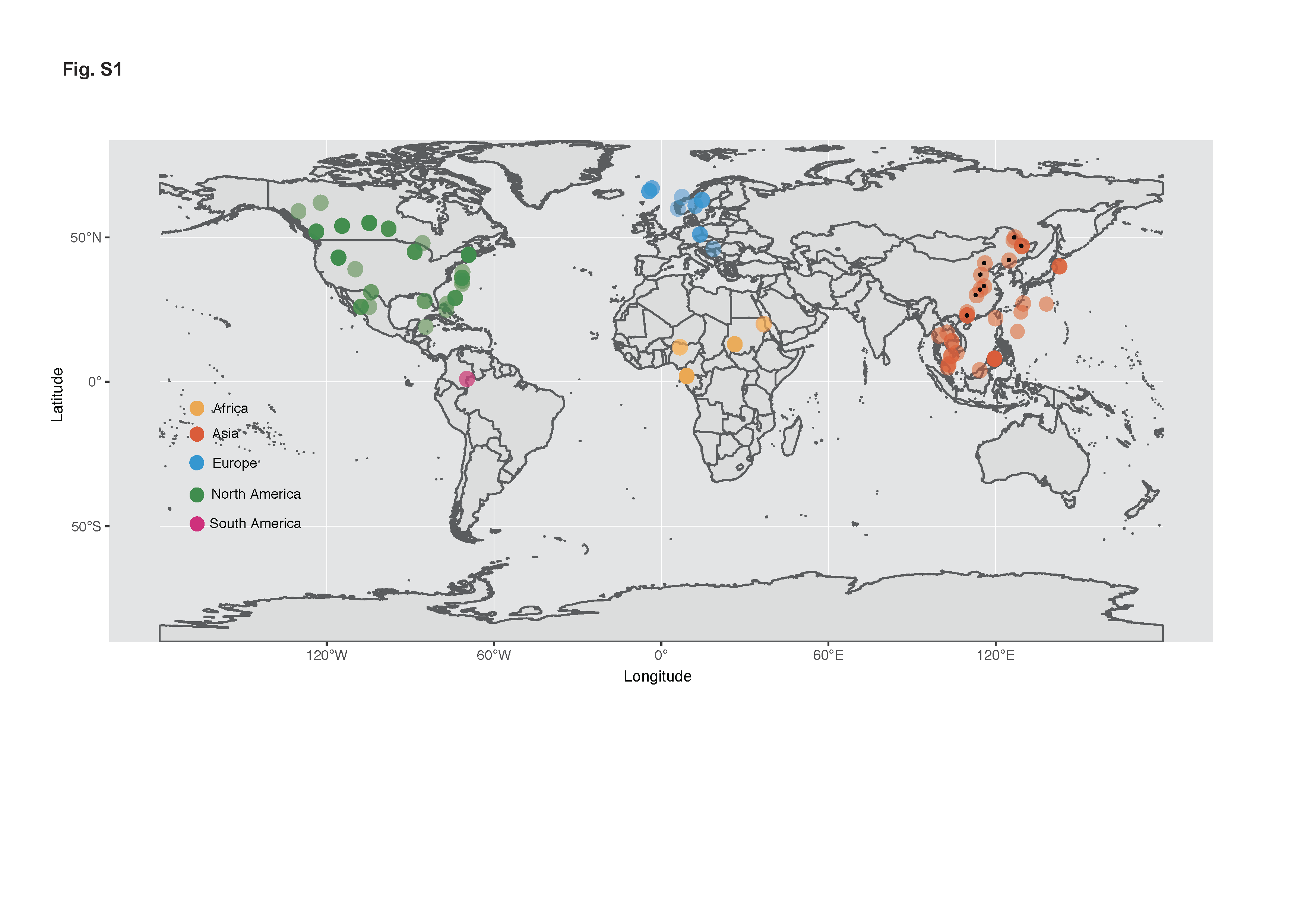

Supplement: FIGURE S1 — Map showing the localities of potato virus Y isolates included in this study. The ggplot2 and maps libraries in R 3.5.1were used to create the map. Novel viral isolates obtained in this study are indicated by black dot. [file Image_1.TIFF]

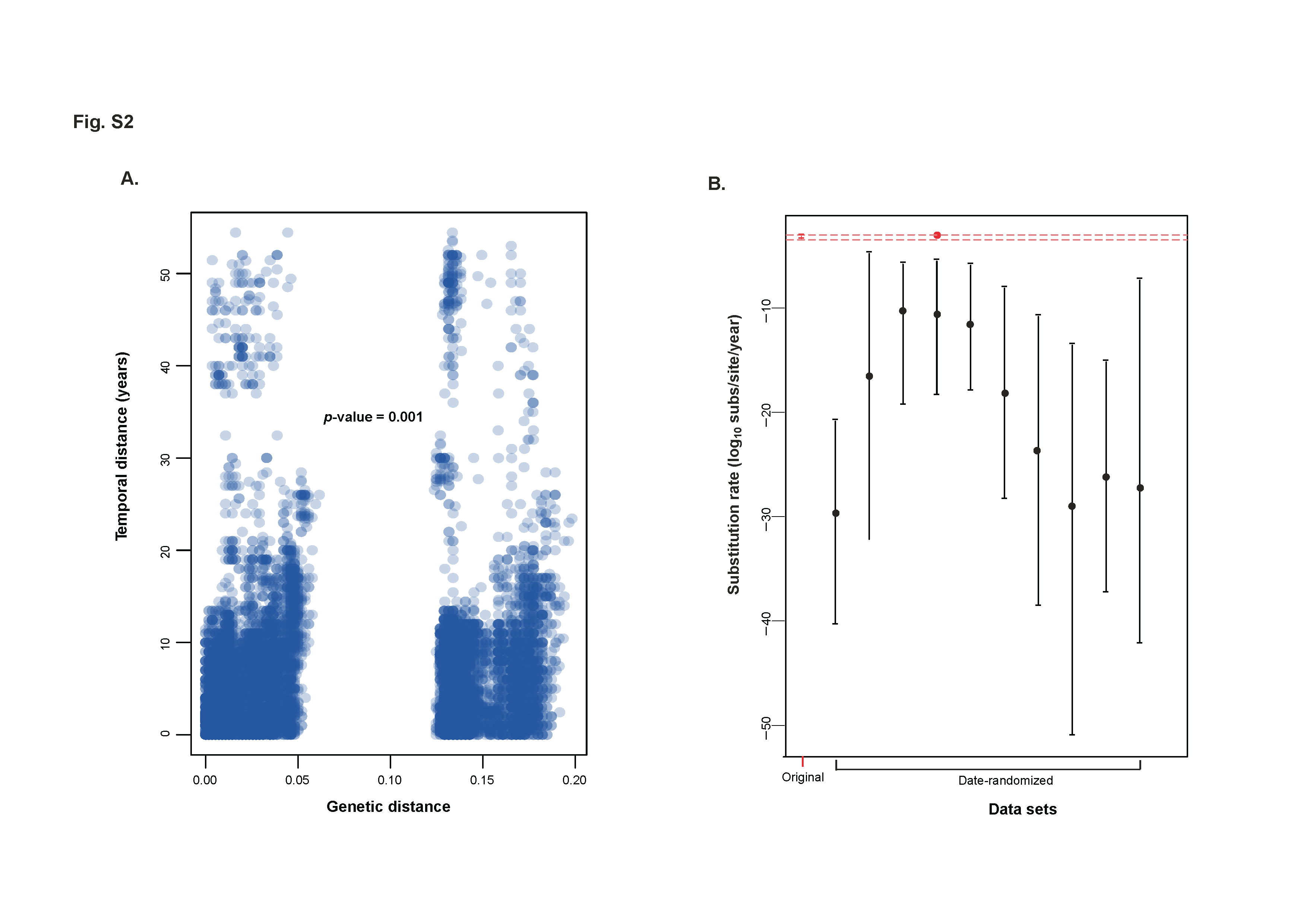

Supplement: FIGURE S2 — Results of tests for the presence of temporal signal in the sequence data. (A) Mantel test of confounding of genetic and temporal distances. The y-axis shows differences in sampling years, and the x-axis indicates genetic distance. (B) Date-randomization test using clustered permutations. The y-axis indicates the substitution rate on a log10 scale, and the x-axis shows the actual data set and 10 different clustered permutations of the dates in the data set. The dashed red lines indicate the 95% credibility interval of the rate estimate from the actual data set. [file Image_2.TIFF]

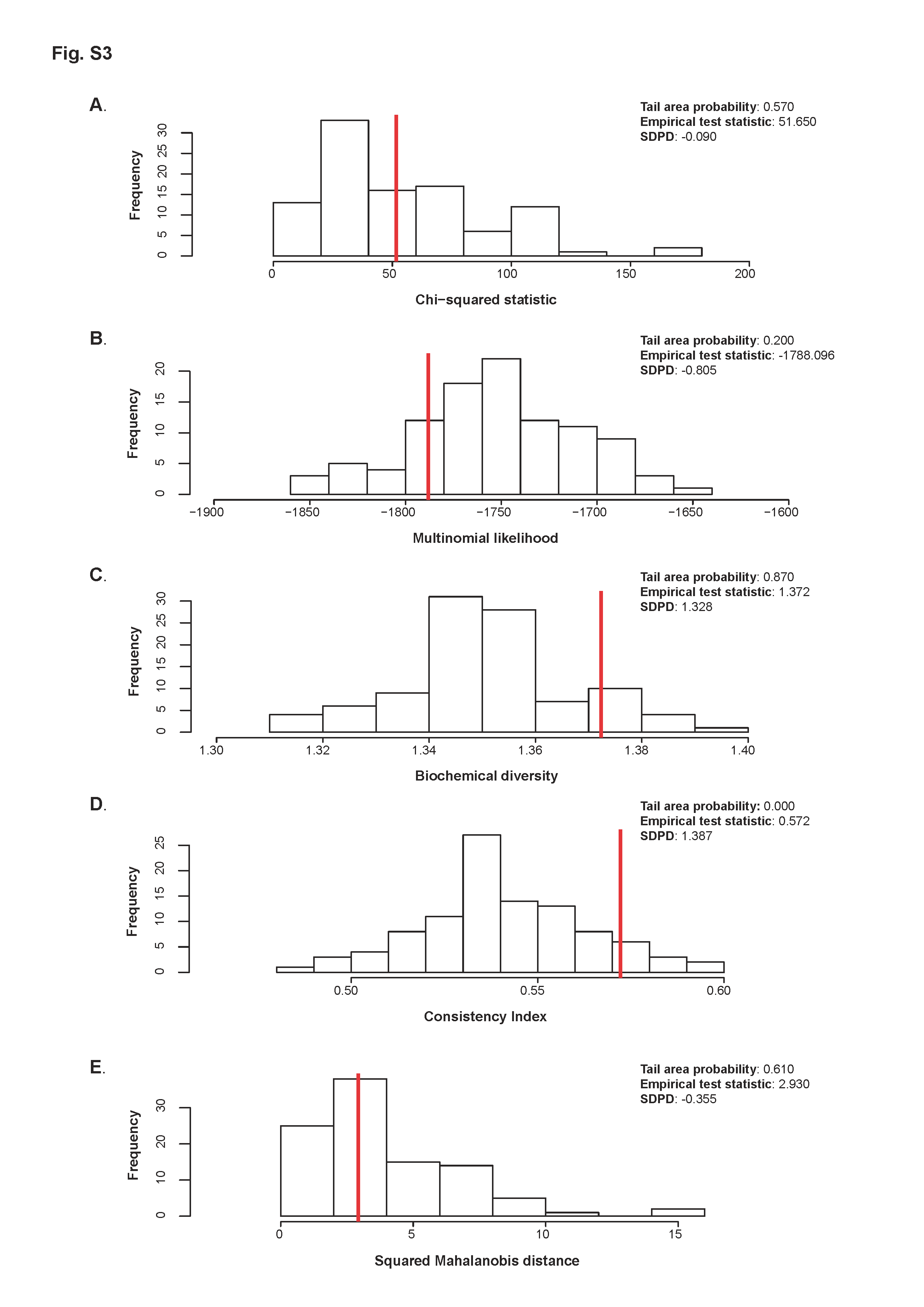

Supplement: FIGURE S3 — Test of substitution model adequacy for the HKY + G4 substitution model, as assessed using five test statistics available in PhyMad: (A) chi-squared statistic, (B) multinomial likelihood, (C) biochemical diversity, (D) consistency index, and (E) squared Mahalanobis distance based on the first four statistics. The histograms of the values of the test statistics are calculated from simulation data set, with the red line indicating the value for the empirical data set. The tail-area probability, empirical test statistic, and standard deviations of the predictive distribution (SDPD) are shown in the top-right of each panel. [file Image_3.TIFF]

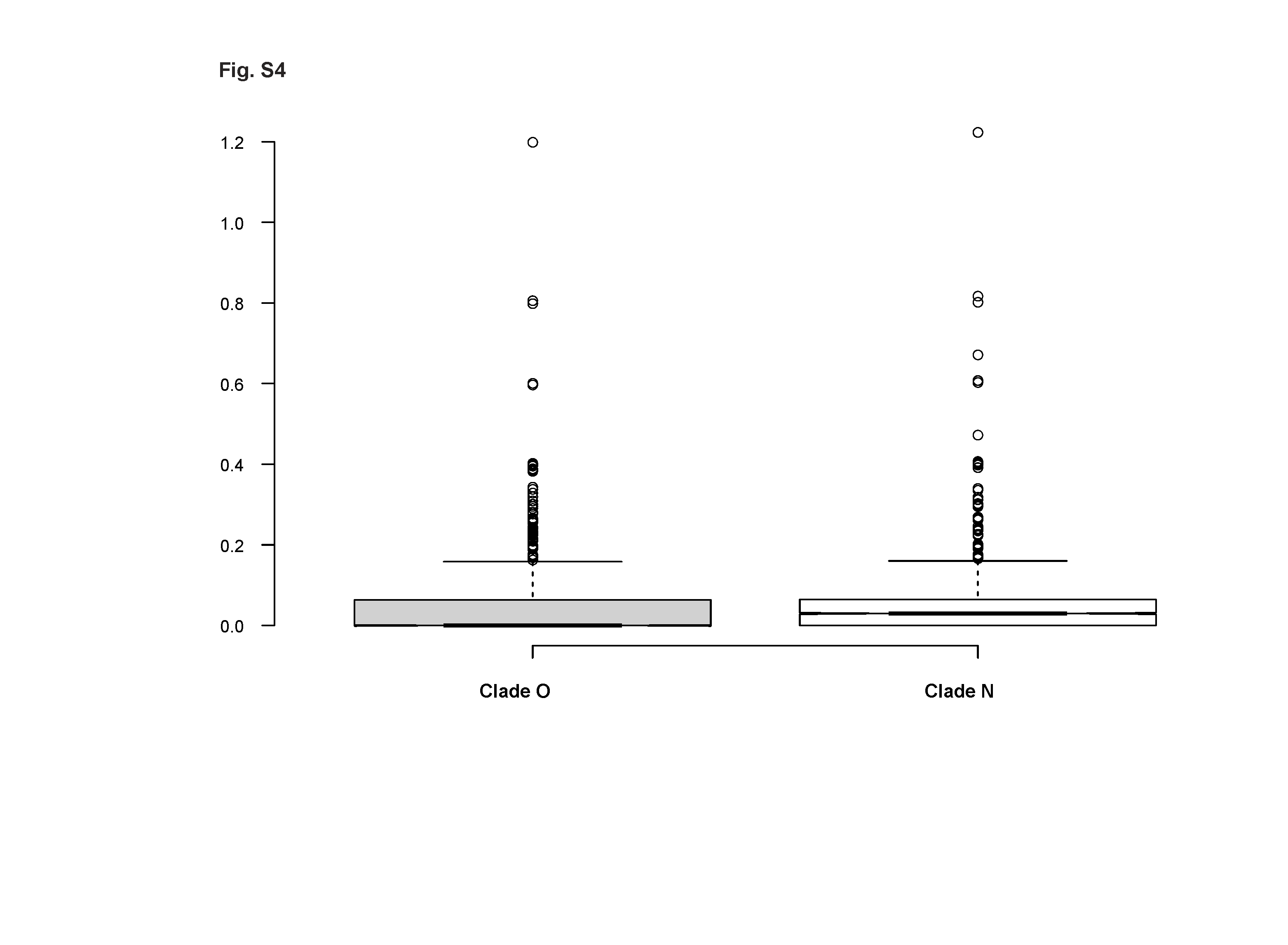

Supplement: FIGURE S4 — Comparison of dN/dS values between the two clades. Boxplots showing the dN/dS ratio of clade O and clade N for the VPg gene. [file Image_4.TIFF]

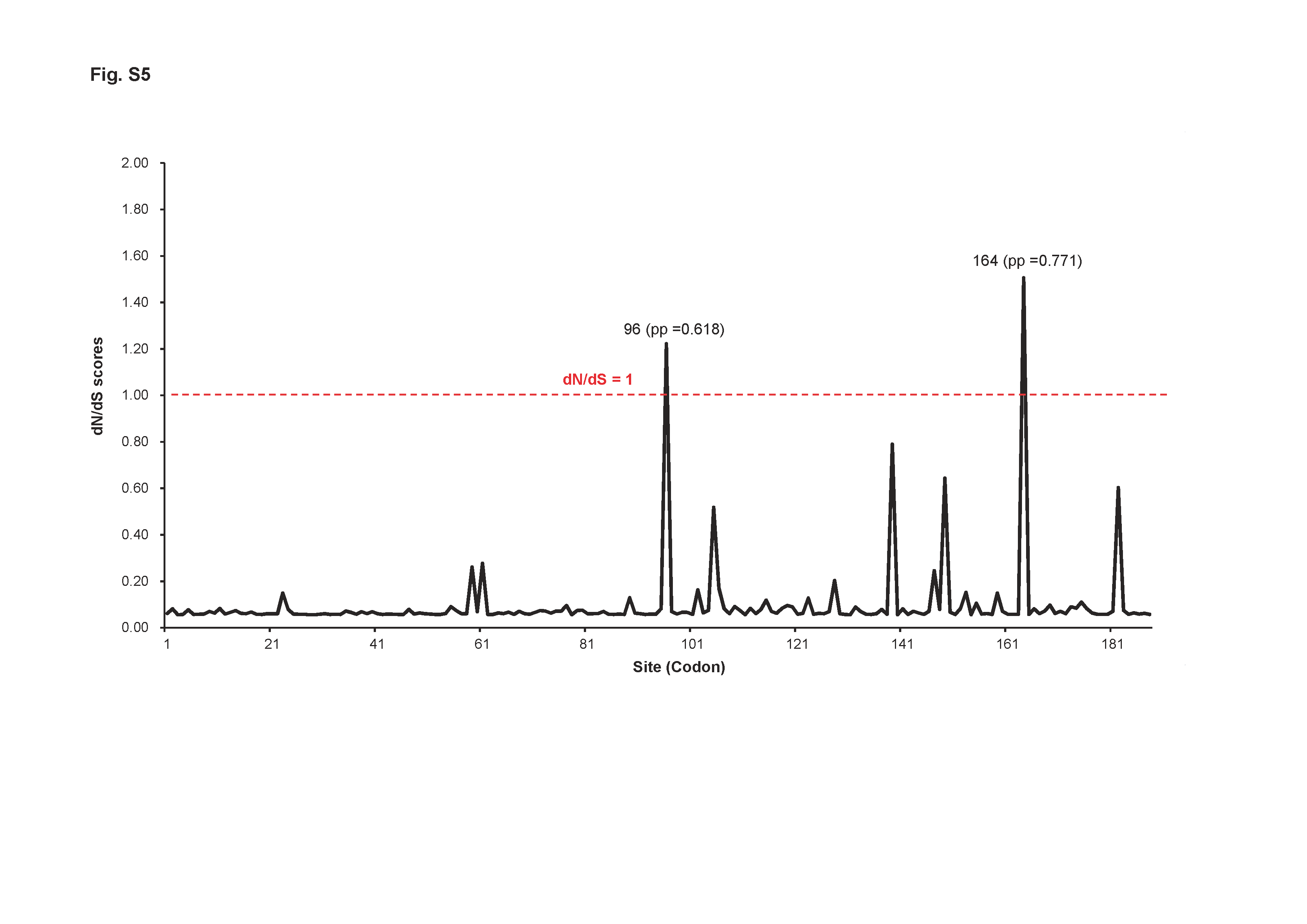

Supplement: FIGURE S5 — Sliding window plot of dN/dS ratios for the VPg gene. Sites under neutral (dN/dS = 1) are indicated in red dotted line. The window size is 20 codons, and the offset between windows is one codon. [file Image_5.TIFF]
