# Supplementary material for: Molecular Evolutionary Analysis of Potato Virus Y Infecting Potato Based on the VPg Gene
Source: Front Microbiol. 2019 Jul 26;10:1708. doi: 10.3389/fmicb.2019.01708 (PMC6676787; doi:10.3389/fmicb.2019.01708)
Supplement: TABLE S1 — Isolates of potato virus Y used in this study. [file Table_1.docx]

**Table S1 Isolates of potato virus Y used in this study**

| **Isolate** | **Country** | **Host**  **origin** | **Collection**  **date** | **Accession number** | **Strain/Genotype** |
| --- | --- | --- | --- | --- | --- |
| NTND6 | Japan | Potato | 1995 | AB331515 | NA-N |
| NTNOK105 | Japan | Potato | 1999 | AB331516 | NA-N |
| NTNHO90 | Japan | Potato | 1998 | AB331517 | NA-N |
| NTNNN99 | Japan | Potato | 1997 | AB331518 | NA-N |
| NTNON92 | Japan | Potato | 1998 | AB331519 | NA-N |
| PVY-27 | Syria | Potato | 2002 | AB461450 | NTN |
| OH | Japan | Potato | 2009 | AB714134 | O |
| T13 | Japan | Potato | 1989 | AB714135 | Unclassified |
| SASA 207 | UK | Potato | 2003 | AJ584851 | N: O |
| SASA-110 | UK | Potato | 2003 | AJ585195 | O |
| SCRI-O | UK | Potato | 2003 | AJ585196 | O |
| SCRI-N | UK | Potato | 2003 | AJ585197 | Unclassified |
| SASA-61 | UK | Potato | 2003 | AJ585198 | NA-N |
| NIB-NTN | Slovenia | Potato | 2003 | AJ585342 | NTN |
| Tu 660 | Canada | Potato | 2002 | AY166866 | NA-N |
| N-Jg | Canada | Potato | 2002 | AY166867 | NA-N |
| 423-3 | USA | Potato | 2005 | AY884982 | NTN |
| RRA-1 | USA | Potato | 2005 | AY884984 | NA-N |
| Alt | USA | Potato | 2005 | AY884985 | N: O |
| PN10A | USA | Potato | 2005 | DQ008213 | N-Wi |
| OR-1 | USA | Potato | 2005 | DQ157179 | N: O |
| NE-11 | USA | Potato | 2005 | DQ157180 | NE-11 |
| PVY-Oz | USA | Potato | 2006 | EF026074 | O |
| PB312 | USA | Potato | 2006 | EF026075 | NTN |
| PB209 | USA | Potato | 2006 | EF026076 | N: O |
| MV99 | Germany | Potato | 2010-09-15 | HE608963 | N-Wi |
| MV175 | Germany | Potato | 2010-09-15 | HE608964 | N-Wi |
| 09-14a | Poland | Potato | 2009 | JF804792 | Unknown |
| YE Epo | Poland | Potato | 1966 | JF804798 | Unknown |
| YO Epo | Poland | Potato | 1963 | JF804799 | Unknown |
| NN300_41 | South Africa | Potato | 2005-07-20 | JN936422 | Unknown |
| PVYNTN23_1 | South Africa | Potato | 2007-09-11 | JN936430 | Unknown |
| SS302_3 | South Africa | Potato | 2010-01-13 | JN936435 | Unknown |
| SS607_36 | South Africa | Potato | 2010-02-12 | JN936437 | Unknown |
| Z001 | South Africa | Potato | 2010-01-05 | JN936439 | Unknown |
| YO-ANT25 | Brazil | Potato | 1985 | JQ924285 | O |
| ALF-VI | Brazil | Potato | 2007 | JQ924287 | NTN |
| M3 | Mexico | Potato | 2009-08-20 | KF850513 | Unknown |
| GF_YL20 | China | Potato | 2011-08-13 | KJ634023 | Unknown |
| ShX14 | China | Potato | 2011-08-13 | KJ634024 | NTN-NW |
| CF_YL21 | China | Potato | 2011-08-13 | KJ801915 | N-Wi |
| 3D | Serbia | Potato | 2013 | KJ946936 | Unknown |
| NTN | Slovenia | Potato | 2007 | KM396648 | NTN |
| CL201501 | China | Potato | 2015-02-04 | KX376950 | NTN-NW |
| CL201502 | China | Potato | 2015-02-04 | KX376951 | NTN-NW |
| CL201503 | China | Potato | 2015-02-04 | KX376952 | NTN-NW |
| CL201504 | China | Potato | 2015-02-04 | KX376953 | NTN-NW |
| CL201505 | China | Potato | 2015-02-04 | KX376954 | NTN-NW |
| CL201507 | China | Potato | 2015-02-04 | KX376955 | Unknown |
| CL201508 | China | Potato | 2015-02-04 | KX376956 | Unknown |
| FQ201501 | China | Potato | 2015-02-04 | KX376957 | N-Wi |
| FQ201502 | China | Potato | 2015-02-04 | KX376958 | NTN-NW |
| FQ201503 | China | Potato | 2015-02-04 | KX376959 | Unknown |
| FQ201504 | China | Potato | 2015-02-04 | KX376960 | Unknown |
| FQ201506 | China | Potato | 2015-02-04 | KX376961 | NTN-NW |
| FQ201507 | China | Potato | 2015-02-04 | KX376962 | Unknown |
| FQ201508 | China | Potato | 2015-02-04 | KX376963 | E |
| FQ201509 | China | Potato | 2015-02-04 | KX376964 | NTN-NW |
| FQ201511 | China | Potato | 2015-02-04 | KX376965 | NTN-NW |
| FQ201512 | China | Potato | 2015-02-04 | KX376966 | NTN-NW |
| FQ201513 | China | Potato | 2015-02-04 | KX376967 | NTN-NW |
| MOD1 | South Africa | Potato | 2013 | KX710153 | Unknown |
| SneP3 | South Africa | Potato | 2013 | KX710154 | Unknown |
| NTN | Slovenia | Potato | 2015-01-19 | KX856986 | NTN |
| ID1_4_32B | USA | Potato | 2004 | KY847939 | O5 |
| ID1_3_11B | USA | Potato | 2004 | KY847943 | NTN |
| ID11_13_11b | USA | Potato | 2004 | KY847944 | NTN |
| ID11_13_12A | USA | Potato | 2004 | KY847945 | N-Wi |
| ID12_102IC3 | USA | Potato | 2012 | KY847946 | NTN |
| ID12_110Ban1 | USA | Potato | 2012 | KY847947 | O5 |
| ID12_22RN8 | USA | Potato | 2012 | KY847948 | NTN |
| ID125 | USA | Potato | 2005 | KY847950 | N-Wi |
| ID13_148Oth | USA | Potato | 2013 | KY847952 | N-Wi |
| ID26 | USA | Potato | 2005 | KY847955 | NE-11 |
| ID89 | USA | Potato | 2005 | KY847959 | N-Wi |
| Linda14 | Germany | Potato | 2013 | KY847961 | N-Wi |
| ME100007 | USA | Potato | 2010 | KY847966 | O |
| ME100011 | USA | Potato | 2010 | KY847968 | NTN |
| ME100031 | USA | Potato | 2010 | KY847969 | NTN |
| ME200cornell | USA | Potato | 2006 | KY847972 | O5 |
| ME4 | USA | Potato | 2006 | KY847973 | NTN |
| ME81 | USA | Potato | 2006 | KY847974 | N: O |
| MN121 | USA | Potato | 2006 | KY847979 | N: O |
| MN15_G_52 | USA | Potato | 2004 | KY847981 | N-Wi |
| MN21 | USA | Potato | 2005 | KY847982 | N-Wi |
| MN85 | USA | Potato | 2006 | KY847983 | NTN |
| MT100006 | USA | Potato | 2010 | KY847986 | Eu-N |
| MT100017 | USA | Potato | 2010 | KY847988 | Eu-N |
| MT29 | USA | Potato | 2004 | KY847989 | O |
| MT52 | USA | Potato | 2005 | KY847990 | N: O |
| MT63 | USA | Potato | 2004 | KY847991 | O |
| ND100040 | USA | Potato | 2010 | KY847992 | NE-11 |
| ND121 | USA | Potato | 2004 | KY847994 | N: O |
| ND18 | USA | Potato | 2005 | KY847995 | N: O |
| ND23 | USA | Potato | 2006 | KY847997 | Unclassified |
| ND68 | USA | Potato | 2004 | KY848000 | N: O |
| ND71 | USA | Potato | 2004 | KY848001 | N: O |
| NE38 | USA | Potato | 2004 | KY848004 | O |
| NE6 | USA | Potato | 2005 | KY848006 | O |
| NY090004 | USA | Potato | 2009 | KY848007 | N: O |
| NY090029 | USA | Potato | 2009 | KY848008 | NTN |
| NY090031 | USA | Potato | 2009 | KY848009 | O |
| NY100003 | USA | Potato | 2010 | KY848012 | O |
| OR16 | USA | Potato | 2005 | KY848018 | N: O |
| OR2 | USA | Potato | 2006 | KY848019 | O |
| OR20 | USA | Potato | 2004 | KY848020 | O |
| OR3 | USA | Potato | 2005 | KY848021 | N-Wi |
| Pondo4 | Germany | Potato | 2013 | KY848023 | 261-4 |
| SU2 | USA | Potato | 2013 | KY848024 | NE-11 |
| WA316 | USA | Potato | 2009 | KY848025 | NE-11 |
| WI120018 | USA | Potato | 2012 | KY848028 | NE-11 |
| WI120092 | USA | Potato | 2012 | KY848029 | O |
| WI120127 | USA | Potato | 2012 | KY848030 | N: O |
| WI3 | USA | Potato | 2004 | KY848031 | O |
| WY1 | USA | Potato | 2005 | KY848034 | O |
| CO12 | USA | Potato | 2004 | KY848037 | O5 |
| CO275 | USA | Potato | 2005 | KY848040 | O5 |
| CO32 | USA | Potato | 2004 | KY848041 | O5 |
| ME_222_18 | USA | Potato | 2005 | KY848048 | O5 |
| ME_250_20 | USA | Potato | 2004 | KY848050 | O5 |
| ME_323_34 | USA | Potato | 2004 | KY848051 | O |
| Egypt11 | Egypt | Potato | 2014 | KY863548 | 261-4 |
| Egypt7 | Egypt | Potato | 2014 | KY863549 | NTN-NW |
| Egypt24 | Egypt | Potato | 2014 | KY863550 | Unclassified |
| Egypt35 | Egypt | Potato | 2014 | KY863551 | NTN-a |
| HLJ26 | China | Potato | 2012-08-23 | MF134425 | NTN-NW |
| 171_1 | USA | Potato | 2014 | MF624282 | Unknown |
| MT12_Oth291 | USA | Potato | 2012 | MF624284 | Unknown |
| MT12_Oth303 | USA | Potato | 2012 | MF624285 | Unknown |
| Oth15_41 | USA | Potato | 2015 | MF624286 | Unknown |
| Oth2016_153 | USA | Potato | 2016 | MF624288 | Unknown |
| WGO15_204 | USA | Potato | 2015 | MF624290 | Unknown |
| **HeiLJ01** | China | Potato | 2014-06-25 | MK144421 | O |
| **HeiLJ02** | China | Potato | 2012-08-24 | MK144422 | N |
| **HeiLJ03** | China | Potato | 2012-10-19 | MK144423 | N |
| **HeiLJ04** | China | Potato | 2013-04-27 | MK144424 | N |
| **HeiLJ05** | China | Potato | 2013 | MK144425 | N |
| **HeiLJ06** | China | Potato | 2011 | MK144426 | N |
| **HeiLJ07** | China | Potato | 2011 | MK144427 | N |
| **HeiLJ08** | China | Potato | 2013-04-27 | MK144428 | N |
| **HeiLJ09** | China | Potato | 2013-07-15 | MK144429 | O |
| **HeiLJ10** | China | Potato | 2013-04-27 | MK144430 | N |
| **HeiLJ11** | China | Potato | 2012 | MK144431 | O |
| **HeiLJ12** | China | Potato | 2012-02-02 | MK144432 | N |
| **HeiLJ13** | China | Potato | 2012-10-19 | MK144433 | N |
| **HeiLJ14** | China | Potato | 2012-10-19 | MK144434 | N |
| **HeiLJ15** | China | Potato | 2012-10-19 | MK144435 | N |
| **HeiLJ16** | China | Potato | 2012-10-19 | MK144436 | N |
| **HeiLJ17** | China | Potato | 2012-10-19 | MK144437 | N |
| **HeiLJ18** | China | Potato | 2012-02 | MK144438 | N |
| **HeiLJ19** | China | Potato | 2013-04-27 | MK144439 | N |
| **YunN20** | China | Potato | 2014-05-29 | MK144440 | N |
| **YunN21** | China | Potato | 2014-05-29 | MK144441 | N |
| **YunN22** | China | Potato | 2012-07-09 | MK144442 | O |
| **YunN23** | China | Potato | 2012-07-09 | MK144443 | N |
| **YunN24** | China | Potato | 2012-07-09 | MK144444 | N |
| **YunN25** | China | Potato | 2012-07-07 | MK144445 | O |
| **ShanD26** | China | Potato | 2012-07-25 | MK144446 | N |
| **NeiM27** | China | Potato | 2015-07-10 | MK144447 | N |
| **NeiM28** | China | Potato | 2013 | MK144448 | N |
| **NeiM29** | China | Potato | 2014 | MK144449 | N |
| **GuangD30** | China | Potato | 2014 | MK144450 | N |
| **GuiZ31** | China | Potato | 2015 | MK144451 | N |
| **GuiZ32** | China | Potato | 2014-05-30 | MK144452 | N |
| **GuiZ33** | China | Potato | 2015 | MK144453 | N |
| **GuiZ34** | China | Potato | 2015 | MK144454 | N |
| **HeB35** | China | Potato | 2015-08-15 | MK144455 | N |
| **HeB36** | China | Potato | 2013-04-18 | MK144456 | O |
| **ShanX37** | China | Potato | 2014-12-15 | MK144457 | N |
| **JiL38** | China | Potato | 2014-11-05 | MK144458 | N |
| **SiC39** | China | Potato | 2016-08-29 | MK144459 | N |
| **HeB40** | China | Potato | 2016-07-15 | MK144460 | N |
| **HeB41** | China | Potato | 2016-09-01 | MK144461 | N |
| **HeiLJ42** | China | Potato | 2017-07-11 | MK144462 | N |
| **HeiLJ43** | China | Potato | 2017-07-12 | MK144463 | O |
| **HeiLJ44** | China | Potato | 2017-07-12 | MK144464 | O |
| N605 | Switzerland | Potato | 1996 | X97895 | Unknown |

Viral isolates sequenced in this study are indicated in bold font.
