# Supplementary material for: Molecular Evolutionary Analysis of Potato Virus Y Infecting Potato Based on the VPg Gene
Source: Front Microbiol. 2019 Jul 26;10:1708. doi: 10.3389/fmicb.2019.01708 (PMC6676787; doi:10.3389/fmicb.2019.01708)
Supplement: TABLE S2 — Marginal likelihoods of different combinations of clock model and tree prior. [file Table_2.DOCX]

**Table S2** Marginal likelihoods of different combinations of clock model and tree prior

| **Molecular clock model** | **Coalescent tree prior** | **Log marginal likelihood** |
| --- | --- | --- |
| Strict clock | Bayesian skyline | -3949.630 |
| Strict clock | Exponential growth | -3944.100 |
| Strict clock | Constant size | -3947.540 |
| **Uncorrelated lognormal relaxed clock** | **Bayesian skyline** | **-3919.129** |
| Uncorrelated lognormal relaxed clock | Exponential growth | -3926.303 |
| Uncorrelated lognormal relaxed clock | Constant size | -3932.282 |

The best-fitting tree prior and molecular clock model are indicated in bold font.
