# Supplementary material for: Molecular Evolutionary Analysis of Potato Virus Y Infecting Potato Based on the VPg Gene
Source: Front Microbiol. 2019 Jul 26;10:1708. doi: 10.3389/fmicb.2019.01708 (PMC6676787; doi:10.3389/fmicb.2019.01708)
Supplement: TABLE S3 — Selective constraint acting on the VPg gene. [file Table_3.DOCX]

**Table 3** Selective constraint acting on the VPg gene

| **Clade** | **Model** | **Number of parameters** | **Log likelihood score** | **Estimates of parameters** | **LRT *P*-value** |
| --- | --- | --- | --- | --- | --- |
| N | Two-ratio (*ω*_0_, *ω*_1_) | 353 | -2844.864 | *ω*_0_= 0.073 (clade O), *ω*_1_= 0.031(clade N) | 0.036 |
|  | One-ratio (*ω*_0_= *ω*_1_) | 352 | -2847.060 | *ω*_0_= 0.064 |  |
| O | Two-ratio (*ω*_0_, *ω*_1_) | 353 | -2844.672 | *ω*_0_= 0.073 (clade N), *ω*_1_= 0.001 (clade O) | 0.029 |
|  | One-ratio (*ω*_0_= *ω*_1_) | 352 | -2847.060 | *ω*_0_= 0.064 |  |
