# Supplementary material for: Molecular Evolutionary Analysis of Potato Virus Y Infecting Potato Based on the VPg Gene
Source: Front Microbiol. 2019 Jul 26;10:1708. doi: 10.3389/fmicb.2019.01708 (PMC6676787; doi:10.3389/fmicb.2019.01708)
Supplement: TABLE S4 — Results of subsampling-Bayesian tip-association significance testing. [file Table_4.DOCX]

**Table S4** Results of subsampling-Bayesian tip-association significance testing

| **Subsampling** | **Region** | **Observed mean** | **Null mean** | ***p*-value** |
| --- | --- | --- | --- | --- |
| Replicate 1 | Africa | 2.34 | 1.65 | 0.016 |
|  | Asia | 8.01 | 1.71 | 0.020 |
|  | Europe | 1.98 | 1.79 | 0.020 |
|  | North America | 2.34 | 1.73 | 0.028 |
| Replicate 2 | Africa | 2.20 | 1.74 | 0.022 |
|  | Asia | 7.99 | 1.60 | 0.020 |
|  | Europe | 1.70 | 1.74 | 0.024 |
|  | North America | 3.11 | 1.79 | 0.020 |
| Replicate 3 | Africa | 2.34 | 1.72 | 0.016 |
|  | Asia | 8.00 | 1.71 | 0.020 |
|  | Europe | 2.19 | 1.70 | 0.016 |
|  | North America | 3.12 | 1.77 | 0.010 |
| Replicate 4 | Africa | 2.62 | 1.71 | 0.020 |
|  | Asia | 9.02 | 1.72 | 0.020 |
|  | Europe | 2.13 | 1.75 | 0.034 |
|  | North America | 2.26 | 1.69 | 0.024 |
| Replicate 5 | Africa | 2.40 | 1.88 | 0.038 |
|  | Asia | 6.00 | 1.86 | 0.020 |
|  | Europe | 2.13 | 1.86 | 0.034 |
|  | North America | 2.52 | 1.65 | 0.018 |
| Replicate 6 | Africa | 2.26 | 1.71 | 0.028 |
|  | Asia | 6.06 | 1.78 | 0.020 |
|  | Europe | 2.11 | 1.73 | 0.034 |
|  | North America | 3.00 | 1.80 | 0.020 |
| Replicate 7 | Africa | 2.09 | 1.73 | 0.260 |
|  | Asia | 8.99 | 1.78 | 0.020 |
|  | Europe | 2.08 | 1.79 | 0.032 |
|  | North America | 3.03 | 1.72 | 0.020 |
| Replicate 8 | Africa | 3.28 | 1.67 | 0.020 |
|  | Asia | 7.00 | 1.65 | 0.020 |
|  | Europe | 2.10 | 1.62 | 0.016 |
|  | North America | 2.91 | 1.76 | 0.020 |
| Replicate 9 | Africa | 2.20 | 1.66 | 0.022 |
|  | Asia | 7.00 | 1.68 | 0.020 |
|  | Europe | 2.01 | 1.70 | 0.014 |
|  | North America | 4.40 | 1.65 | 0.020 |
| Replicate 10 | Africa | 2.25 | 1.58 | 0.016 |
|  | Asia | 8.00 | 1.68 | 0.020 |
|  | Europe | 1.15 | 1.63 | 0.010 |
|  | North America | 2.75 | 1.65 | 0.040 |
